# Supplementary material for: Strategies to resolve the gap in adolescent tuberculosis care at four health facilities in Uganda: The teenager’s TB pilot project
Source: PLoS One. 2024 Apr 11;19(4):e0286894. doi: 10.1371/journal.pone.0286894 (PMC11008838; doi:10.1371/journal.pone.0286894)
Supplement: S1 File — (PDF) [file pone.0286894.s002.pdf]

TELEPHONE: +256-41554008/1
FAX: +256-414-5325591
E-mail: admin@mulago.or.ug
Website: www.mulago.or.ug

IN ANY CORRESPONDENCE ON THIS
SUBJECT PLEASE QUOTES NO................

MULAGO NATIONAL REFERRAL HOSPITAL
P. O. Box 7051
KAMPALA, UGANDA

20th October 2020.

Dr. Bruce Kirenga
Principal Investigator
Makerere Lung Institute

Dear Bruce,

RE: APPROVAL OF PROTOCOL MHREC 1922: "STRATEGIES FOR RESOLVING THE GAP IN ADOLESCENT TB CARE IN UGANDA: A PILOT CLUSTER RANDOMIZED – CONTROLLED TRIAL – V1.0 July 2020".

The Mulago Hospital Research and Ethics Committee reviewed your proposal referenced above and granted approval of this study on 20th October, 2020. The conduct of this study will therefore run for a period of one (1) year from 20th October, 2020 to 19th October, 2021.

This approval covers the protocol and the accompanying documents listed below;

- Adolescent TB Assessment Tool– V1.0 July 2020
- Participant Information Sheet– V1.0 July 2020
- Consent Form for Enrollment to Participate in a Research Study – V1.0 July 200.
- Parental Consent Form for Enrollment to Participate in a Research Study
- Assent Form for Enrollment to Participate in a Research Study – V1.0 July 2020

This approval is subjected to the following conditions:

1. That the study site may be monitored by the Mulago Hospital Research and Ethics Committee at any time.
2. That you will be abide by the regulations governing research in the country as set by the Ugandan National Council for Science and Technology including abiding to all reporting requirements for serious adverse events, unanticipated events and protocol violations.
3. That no changes to the protocol and study documents will be implemented until they are reviewed and approved by the Mulago Hospital Research and Ethics Committee.
4. That you will submit this approved protocol and all accompanying documents for approval to UNCST before starting the study. In case of studies involving drug and medical devices, approval must be obtained from the National Drug Authority before starting the study
5. That you provide quarterly progressive reports and request for renewal of approval at least 60 days before expiry of the current approval.
6. That you provide an end of study report upon completion of the study including a summary of the results and any publications.
7. That you will include Mulago Hospital in your acknowledgements in all your publications.

I wish you the best in this Endeavour.

Yours sincerely

APPROVED
APPROVAL DATE 20 OCT 2020
EXPIRY DATE 19 OCT 2021
MULAGO RESEARCH ETHICS COMMITTEE
IRC

DR. NAKWAGALA FREDERICK NELSON
CHAIR- MULAGO HOSPITAL RESEARCH & ETHICS COMMITTEE.
