# Supplementary material for: Strategies to resolve the gap in adolescent tuberculosis care at four health facilities in Uganda: The teenager’s TB pilot project
Source: PLoS One. 2024 Apr 11;19(4):e0286894. doi: 10.1371/journal.pone.0286894 (PMC11008838; doi:10.1371/journal.pone.0286894)
Supplement: Attachment — Submitted filename: PLOS ONE Response to reviewers.docx [file pone.0286894.s006.docx]

**PONE-D-23-15403: Responses to the review comments**

Comment: Consider a short title that captures the attention of the reader.

***Response:*** The title has been shortened to read “**Strategies to resolve the gap in Adolescent Tuberculosis care in Uganda: The TEEN TB pilot project”** as indicated in line No. 1&2 on the manuscript

Comment: The abstract is too long.
***Response:*** It has been shortened in the revised manuscript- refer to line Nos. 16 to 49 in the Manuscript

Comment: The background is inadequate and needs strength for its contents.

***Response:*** Additional literature on Adolescents Tuberculosis has been added- refer to the main background on the manuscript- No 51 -81

Comment: Why you have used both the P value and the confidence interval at the same time?

***Response:*** The P Values and confident intervals have been re- arranged in the result section of abstract and main manuscript On line no 35 and 176 respectively in the manuscript

Comment: The manuscript fails to meet the standard guideline of PONE e.g. the study setting came after the study design

***Response:*** The order was changed in the revised Manuscript, i.e., we have the study setting before study design; refer to.line number 83-92 & 93-132 in the Manuscript

Comment: What is the specific study design used?

***Response:*** It was a human centered design- Meaning the adolescents were involved in designing process of the intervention package (i.e. the TB awareness posters, screening cards and the local song) at the initial stage. This is clearly explained in the project design section; line no 94-96 of the manuscript

Comment: Did the diagnostic capacity or positive predictive rate of both Gene Xpert and smear microscopy equal? Why you have used either of the test equally otherwise?

***Response:*** We deleted the word” smear microscopy” because more than three quarters of the samples were tested using Gene Xpert, the few that were tested microscopically were removed during data analysis.

Comment: What type of specific consents was used for those less than 15, 15-18 and greater than 18 years?

***Response:*** We used Assent forms for adolescents’ less than 18 years and Consent forms for those who were 18 years and above. It’s clearly explained in the methodology section; line no 124 in the manuscript.

Comments: How do you differentiate that the adolescents had arrived to the health facility due to your particular intervention?

***Response:*** For the screening cards, the ones taken and returned from the community were tracked using a health facility tracker. For the TB awareness posters and the local song, a TB assessment form was used to find the source of information before they were enrolled to the project.
Comment: What are the specific interventions? What is the dose, schedule, and the information conveyer quality? Means of delivery? Cultural appropriateness and language of the intervention needs detail explanation?

***Response:*** 1- Tuberculosis screening cards with TB case finding questions(English & local language) were used by the TB positive adults to screen their own adolescents at home, any of the adolescents that were found with one or more of the TB symptoms were referred to health facilities for further assessment and care.- No 115 – 119 in the Manuscript

2- Tuberculosis awareness posters (English & local language) were designed and deployed in communities within the catchment areas of the project health facilities. These were mostly placed in locations frequently accessed by adolescent like markets, places of worship and drug shops- No 112 and 113 in the manuscript.

These were deployed one month before the study begun and throughout the study period.

3- The Local song was played from selected radio stations with wider coverage to the targeted communities. The song was played for one month before the study begun and throughout the study period- No 114 and 115 in the manuscript.

Comment: What was done to ensure data quality?

***Response***: A qualified Research team was trained on the study protocol, data collection tools were designed, approved by the ethics committee and pre-tested before data collection. The Kobo collect software was used to collect the data, the data was cleaned and queries resolved before transportation to STATA version 14 for analysis. Please refer to the section on data quality control (line no. 143- 148) incorporated on the revised manuscript

Comment: What does descriptive univariate analysis mean?

***Response:*** This was a mistake in writing; we meant descriptive statistics was used to summarize and describe individual study variables- refer to line no 163 in the revised manuscript

Comment: The results, discussion, conclusion and recommendation need major revision in line with the objective.

***Response***: Revision was done for discussion, conclusion and recommendations sections Limitations were separated from the conclusion; refer to the attached revised manuscript line no. 336, and 429 respectively in the manuscript

Comment: Please clarify the sources of funding (financial or material support) for your study.

***Response:*** The study was funded by Makerere University research and Innovation fund (MAKRIF).

Comment: State what role the funders took in the study.

***Response***: The funders had no role in study design, data collection and analysis, decision to publish, or preparation of the manuscript.

Comment: If any authors received a salary from any of your funders, please state.

***Response:*** None of the authors received a salary from the funders.

Comment: In your Data availability statement, you have not specified where the minimal data set underlying the results described in your manuscript can be found?

***Response:*** All relevant data and the supporting information files have been provided

Comment: If you did not receive any funding for this study, please state

***Response:*** The authors received funding for carrying out research, but no financial support to authorship and publication of this manuscript.

NB. All minor comments (structure, grammar punctuations), and all suggestions by the editor and reviewers were catered for and reflected in the revised manuscript.
