# Supplementary material for: Strategies to resolve the gap in adolescent tuberculosis care at four health facilities in Uganda: The teenager’s TB pilot project
Source: PLoS One. 2024 Apr 11;19(4):e0286894. doi: 10.1371/journal.pone.0286894 (PMC11008838; doi:10.1371/journal.pone.0286894)
Supplement: Attachment — Submitted filename: Feed back_zg Dememew.docx [file pone.0286894.s007.docx]

**Title:** replace ‘Adolescent Tuberculosis’ with ‘adolescent tuberculosis’

**Abstract**

You may omit the headings-- introduction, methodology, results, and conclusions.

Please write the full name of TEEN

The design is before after or quasi experimental. Human centered could be the framework used to guide the study. Check this in the body/method section.

“There was a minimal increase in the average monthly number of adolescents with a positive result from 1.6 to 2.4 and linkage to TB care services from 2 to 3.1. These were not statistically significant at p=0.170 and p=0.154 respectively.” Could be replaced with “There was a minimal increase in the average monthly number of adolescents with a positive result from 1.6 (p=0.170) to 2.4 and linkage to TB care services from 2 to 3.1 (p=0.154)”

**Introduction**

Can you make the data source of TB epidemiology of global, Africa and Uganda same years—preferably 2022 global TB rpeort.

Methods

Note: the study/research design is before after or quasi experiment while the framework or a study approach is human centered design (HCD).

\# less than 10 could be written in words e.g 4- four, 8=eight etc No need to write like three (3), four (4), six (6)…

Please avoid capital letters in the middle of the sentine e.g ‘’The training focused on Four (4) key interventions…’’ can be written as ‘‘’The training focused on four key interventions…’’

Check the consistence of clinical criteria.

“… those who presented with any of the four symptoms (cough, fever, drenching night sweats and weight loss) were consented (≥ 18years), or assented (< 18 years) and enrolled into the project.”

AND

“… predefined according to the World Health Organisation’s (WHO) criteria (cough for ≥2 weeks; persistent fever for ≥2 weeks; noticeable weight loss; and excessive night sweats).”

Write the full name of OPD. As first appeared all abbreviations should be written in full. E.g Anti-retroviral therapy (ART), outpatient department (OPD)….

Replace ‘’ Data management/analysis’ with ‘’ Data management and analysis’’.

‘Ethics approvals and informed consent’ is usually written as ‘Ethical consideration or Ethical review”. Please check the the PLOS ONE journal criteria.

Result

Please re-check punctuation. Example

‘’ . Majority, 198 (50%) were aged between 10-15 years, they were mainly females 255(65%), and still in school 298(76%). Majority, 336(85%) were unmarried. Kawolo hospital enrolled the highest number, 137(35%’’ could be written as

‘’ Majority, 198 (50%), were aged between 10-15 years.They were mainly females 255 (65%), and still in school 298(76%). The majority, 336(85%), were unmarried. Kawolo hospital enrolled the highest number, 137(35%’’

Table 1:

Check fi the age group include 20 years

Up to 330 (84%) can be written as ‘‘Three hundred thirty (84%)…’’

Up to 49 (68%) can be written as ‘’ Fourth nine (84%)…’’

You may delete ‘’ The impact of the package on adolescent TB care is presented along the TB care cascade In Figure 1, 2, Tables 3, 4 and 5. The entire analysis revealed the following results:’’

**Reference**: please correct per the PLOS ONE style throughout- not in superscript^1^ but in bracket [1]. The list of references should be per the PLOS style, Vancouver
