# Supplementary material for: Strategies to resolve the gap in adolescent tuberculosis care at four health facilities in Uganda: The teenager’s TB pilot project
Source: PLoS One. 2024 Apr 11;19(4):e0286894. doi: 10.1371/journal.pone.0286894 (PMC11008838; doi:10.1371/journal.pone.0286894)
Supplement: Attachment — Submitted filename: Response to reviewers.docx [file pone.0286894.s008.docx]

**PONE-D-23-15403R1: Response to reviewer’s comments.**

**Comment:** Title: replace ‘Adolescent Tuberculosis’ with ‘adolescent tuberculosis’

***Response:*** The replacement was done as indicated on line number **1** of the manuscript.

**Comment:** Abstract; you may omit the headings-- introduction, methodology, results, and conclusions.

***Response:*** These heads were removed; refer to the abstract on line numbers **17-47** of the manuscript.

**Comment:** Please write the full name of TEEN

***Response:*** The full name for ‘TEEN’’ is “teenager’s” as indicated on line number **2** and **422**(acknowledgement) of the manuscript.

**Comment:** Methods; Note: the study/research design is before and after or quasi experiments while the framework or a study approach is human centered design (HCD).

***Response:*** The sentences were re-written as indicated on line numbers **23 &27**(**abstract**) and **93 & 96-97** (**project design**) - refer to the manuscript.

**Comment:** “There was a minimal increase in the average monthly number of adolescents with a positive result from 1.6 to 2.4 and linkage to TB care services from 2 to 3.1. These were not statistically significant at p=0.170 and p=0.154 respectively.” Could be replaced with “There was a minimal increase in the average monthly number of adolescents with a positive result from 1.6 to 2.4(p=0.170) and linkage to TB care services from 2 to 3.1 (p=0.154)”

***Response:*** The correction was made as indicated in line numbers **43 & 44** of the manuscript

**Comment:** Introduction- Can you make the data source of TB epidemiology of global, Africa and Uganda same years—preferably 2022 global TB report.

***Response:*** The Global, African and Ugandan TB epidemiology data for adolescents aged five to fourteen years was considered for the same year (2021) as reflected in 2022 global report. This is indicated on line numbers **53 to 61** of the manuscript.

**Comment:** # less than 10 could be written in words e.g. 4- four, 8=eight etc. No need to write like three (3), four (4), and six (6)…

***Response:*** The numbers were written in words thus; fourteen, in line number **19,** four as shown on line numbers **99** , three as indicated in line number **106 & 121,** six on line **120** of the manuscript.

**Comment:** Please avoid capital letters in the middle of the sentence e.g ‘’The training focused on Four (4) key interventions…’’ can be written as ‘‘’The training focused on four key interventions…’’

***Response:*** This was corrected as the training focused on four key ingredients, as indicated in line number **107** of the manuscript.

**Comment:** Check the consistence of clinical criteria.“… those who presented with any of the four symptoms (cough, fever, drenching night sweats and weight loss) were consented (≥ 18years), or assented (< 18 years) and enrolled into the project.”

AND

“… Predefined according to the World Health Organization’s (WHO) criteria (cough for ≥2 weeks; persistent fever for ≥2 weeks; noticeable weight loss; and excessive night sweats).”

***Response:*** The sentence was re-written as; “Those who presented with predefined tuberculosis symptoms according to the World Health Organization’s (WHO) criteria (cough for ≥2 weeks; persistent fever for ≥2 weeks; noticeable unintentional weight loss; and excessive night sweats), were consented if they were eighteen years or more, or assented if less than eighteen years. All those who consented or assented were enrolled into the project.”

Refer to line numbers **122 to 127** of the manuscript.

**Comment:** Write the full name of OPD. As first appeared all abbreviations should be written in full. E.g. Anti-retroviral therapy (ART), outpatient department (OPD)….

***Response:*** These have been corrected and written in full names; please refer to line number **137 & 138** in the manuscript.

**Comment:** Replace ‘’ Data management/analysis’ with ‘’ Data management and analysis’’.

***Response:*** “Data management /analysis procedure” was written as” Data management and analysis”, refer to line number **164** in the manuscript.

**Comment:** ‘Ethics approvals and informed consent’ is usually written as ‘Ethical consideration or Ethical review”. Please check the the PLOS ONE journal criteria.

***Response:*** ‘Ethics approvals and informed consent’, was re- written as “Ethical considerations”, “Ethical approvals”, was re- written as “The research project ethical approvals”; Please refer to line number **172 & 173** respectively in the manuscript.

**Comment:** Result; Please re-check punctuation. Example

‘’Majority, 198 (50%) were aged between 10-15 years, they were mainly females 255(65%), and still in school 298(76%). Majority, 336(85%) were unmarried. Kawolo hospital enrolled the highest number, 137(35%)’’ could be written as ‘’ Majority, 198 (50%), were aged between 10-15 years. They were mainly females 255 (65%), and still in school 298(76%). The majority, 336(85%), were unmarried. Kawolo hospital enrolled the highest number, 137(35%’’

***Response:*** Corrections were made on punctuation; as indicated on line numbers **184** - **187** in the manuscript.

**Comment:** Table 1: Check for the age group, includes 20 years

***Response:*** The age group (16- 20years) on table 1 was corrected to (16- 19 years). Refer to table 1 on line number **191** in the manuscript.

**Comment:** Up to 330 (84%) can be written as ‘‘Three hundred thirty (84%)…’’

***Response:*** The sentence was reframed as sixty four (16%) of adolescents had never heard about TB, as indicated on number **196** of the manuscript.

**Comment:** Up to 49 (68%) can be written as ‘’ Forty nine (68%)…’’

***Response:*** The sentence was re-written as shown on line number **196** of the manuscript. All other sentences with similar issues were re-written as presented on line numbers **196- 203.**

**Comment:** You may delete ‘’ The impact of the package on adolescent TB care is presented along the TB care cascade In Figure 1, 2, Tables 3, 4 and 5. The entire analysis revealed the following results:’’

***Response:* Response:** The sentences were deleted as indicated on line numbers **254, 255 & 256** of the tracked manuscript. In the manuscript, line number **209** has the heading **“Impact of package on TB care cascade”,** indicated after the rest of the words were deleted.

**Comment:** Reference: please correct per the PLOS ONE style throughout- not in superscript1 but in bracket [1]. The list of references should be per the PLOS style, Vancouver.

***Response:*** The references that were written as superscript have been written in bracket format in line with Vancouver style, refer to line numbers **52,53,57,59,61,63.65,68,70,74,and 76 (Background)**, and **348, 351, 356, 360, 370, 374, 379, 386, 395, and 399(Discussion)** in the manuscript.

**NB**. 1- The former references (no. 2 **and 3)** were deleted; reference numbers from **4 to 19** in the tracked manuscript were replaced with **2 to 17** respectively as indicated in the manuscript.

**NB.** 2- Page numbering was revised in the tracked manuscript and manuscript.

**NB**. 3- Minor corrections, e.g. on punctuations, spellings, wording, etc., have been attended to in the tracked manuscript and the manuscript.
